# Pharmacokinetics, safety, and efficacy of 20% subcutaneous immunoglobulin (Ig20Gly) administered weekly or every 2 weeks in Japanese patients with primary immunodeficiency diseases: a phase 3, open-label study

**DOI:** 10.1093/immadv/ltae001

**Published:** 2024-03-01

**Authors:** Hirokazu Kanegane, Akifumi Endo, Satoshi Okada, Hidenori Ohnishi, Masataka Ishimura, Ryuta Nishikomori, Kohsuke Imai, Shigeaki Nonoyama, Hideki Muramatsu, Taizo Wada, Atsushi Kuga, Ko Sakamoto, Sharon Russo-Schwarzbaum, Liang-Hui Chu, Barbara McCoy, Zhaoyang Li, Leman Yel

**Affiliations:** Department of Child Health and Development, Tokyo Medical and Dental University, Tokyo, Japan; Department of Child Health and Development, Tokyo Medical and Dental University, Tokyo, Japan; Department of Pediatrics, Hiroshima University Graduate School of Biomedical and Health Sciences, Hiroshima, Japan; Graduate School of Medicine, Gifu University, Gifu, Japan; Department of Pediatrics, Kyushu University Hospital, Fukuoka, Japan; Department of Pediatrics and Child Health, Kurume University Hospital, Fukuoka, Japan; Department of Pediatrics, National Defense Medical College, Saitama, Japan; Department of Pediatrics, National Defense Medical College, Saitama, Japan; Department of Pediatrics, Nagoya University Hospital, Aichi, Japan; Institute of Medical, Pharmaceutical and Health Sciences, Kanazawa University Hospital, Ishikawa, Japan; Takeda Pharmaceutical Company Limited, Osaka, Japan; Takeda Pharmaceutical Company Limited, Osaka, Japan; Baxalta Innovations GmbH, a Takeda company, Vienna, Austria; Takeda Development Center Americas, Inc., Cambridge, MA, USA; Baxalta Innovations GmbH, a Takeda company, Vienna, Austria; Takeda Development Center Americas, Inc., Cambridge, MA, USA; Takeda Development Center Americas, Inc., Cambridge, MA, USA

**Keywords:** immunoglobulin replacement therapy, inborn errors of immunity, primary immunodeficiencies, subcutaneous immunoglobulin

## Abstract

This phase 3, open-label, multidose study (NCT04346108) evaluated the pharmacokinetics, safety, tolerability, and efficacy of immunoglobulin subcutaneous (human) 20% solution (Ig20Gly) administered weekly and every 2 weeks in Japanese patients with primary immunodeficiency diseases (PIDs). The study was conducted at eight study sites in Japan and enrolled patients aged ≥2 years with PIDs treated using a stable intravenous immunoglobulin dose for ≥3 months prior to the study. Patients received intravenous immunoglobulin every 3 or 4 weeks at pre-study dose (200–600 mg/kg) for 13 weeks (Epoch 1), subcutaneous Ig20Gly (50–200 mg/kg) once weekly for 24 weeks (Epoch 2), and Ig20Gly (100–400 mg/kg) every 2 weeks for 12 weeks (Epoch 3). The primary endpoint was serum total immunoglobulin G (IgG) trough levels during Epochs 2 and 3. Overall, 17 patients were enrolled (median [range] age: 24 [5–69] years; 59% male) and participated in Epochs 1 and 2; seven patients entered Epoch 3. Serum total IgG trough levels were maintained at >8 g/l: geometric means (95% confidence intervals) at the end of Epochs 2 and 3 were 8.56 (8.03–9.12) g/l and 8.39 (7.89–8.91) g/l, respectively. Related treatment-emergent adverse events were all mild in severity; the most common treatment-emergent adverse events (excluding infections) in Epochs 2 and 3 were injection site swelling (24%) and injection site erythema (18%). This is the first trial to demonstrate the efficacy and favourable safety profile of 20% subcutaneous immunoglobulin administered every 2 weeks in adult and paediatric Japanese patients with PIDs.

## Graphical Abstract

**Graphical Abstract:**
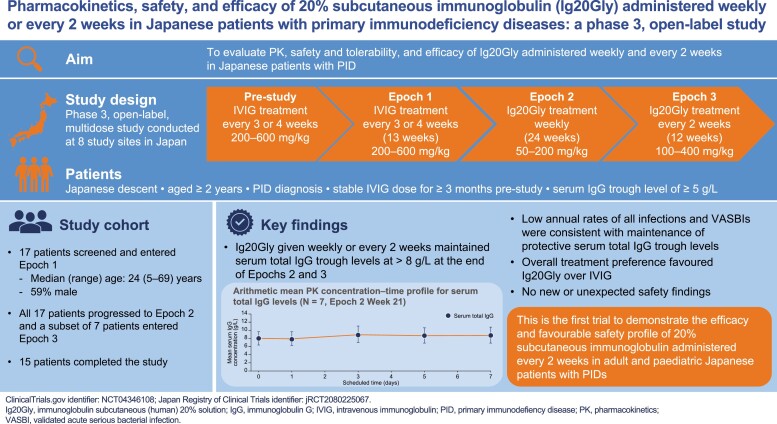

## Introduction

Primary immunodeficiency diseases (PIDs), also referred to as inborn errors of immunity [1], are a group of diseases that result in increased susceptibility to recurrent infections secondary to the underlying defects in adaptive and/or innate immune system [2, 3]. In a 2018 Japanese nationwide survey of 1307 participants, the prevalence of PID/inborn errors of immunity was estimated to be 2.2 patients per 100 000 population, with antibody deficiencies reported by 24% of respondents [4]. The mainstay of treatment for patients with PIDs unable to generate and/or maintain an effective antibody response is immunoglobulin replacement therapy (IgRT), which can be administered through an intravenous or subcutaneous route [3, 5]. Subcutaneous immunoglobulin (SCIG) does not require venous access, can be self-administered at home, and has been shown to be as effective as intravenous immunoglobulin (IVIG) at preventing infections in patients with PIDs, with fewer systemic adverse reactions [6, 7]. However, SCIG infusions are conventionally administered in smaller volumes than IVIG, necessitating more frequent administration than IVIG and a larger number of infusion sites [6, 8].

Immune globulin subcutaneous (human) 20% solution (Ig20Gly; Cuvitru; Baxalta US Inc., a member of the Takeda group of companies, Lexington, MA, USA) is a SCIG approved for the treatment of PIDs in 37 countries. In the USA, Ig20Gly is approved as an IgRT for PIDs in adult and paediatric patients aged 2 years and older [9], and in Europe for the treatment of PIDs with impaired antibody production and certain secondary immunodeficiency syndromes in adults, and children and adolescents (aged 0–18 years) [10]. Ig20Gly is a highly concentrated immunoglobulin G (IgG) formulation that can be administered using smaller total infusion volumes and at higher rates than less-concentrated SCIG products [6, 11, 12]. Until recently, other SCIG 20% formulations available for the treatment of PIDs in Japan have been limited to weekly infusions; only recently has administration every 2 weeks been approved (April 2023) [13]. With Ig20Gly, there is the option to administer infusions at regular intervals from daily to every 2 weeks [9, 10], with efficacy, safety, and tolerability demonstrated in adult and paediatric immunoglobulin-experienced patients with PID in the two pivotal phase 2/3 clinical trials in Europe (NCT01412385) [12] and North America (NCT01218438) [11]. The findings of these pivotal studies have been supported in post-authorization, real-world studies, which highlight the flexibility and tolerability of Ig20Gly in paediatric and adult patients with PIDs [14–17]. The study reported herein was conducted to evaluate the pharmacokinetics (PK), safety and tolerability, and efficacy of Ig20Gly administered weekly and every 2 weeks in Japanese patients with PIDs. Ig20Gly is the first 20% SCIG formulation using glycine as a stabilization and buffering agent to be studied in Japanese patients.

## Methods and materials

### Study design

**Figure 1.**
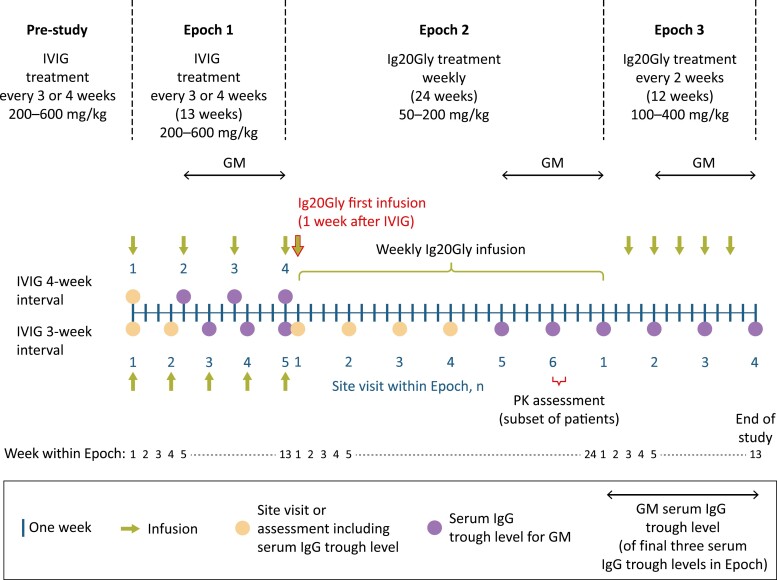
Study design schematic. GM, geometric mean; Ig20Gly, immune globulin subcutaneous (human) 20% solution; IgG, immunoglobulin G; IVIG, intravenous immunoglobulin; PK, pharmacokinetic.

This was a phase 3, open-label, multidose study (ClinicalTrials.gov identifier: NCT04346108; Japan Registry of Clinical Trials identifier: jRCT2080225067) conducted at eight study sites in Japan. The study was comprised of three epochs (Fig. 1). Eligible patients were initially entered into Epoch 1 to receive standard-of-care IVIG infusions at the pre-study dose of approximately 200–600 mg/kg body weight every 3 weeks or every 4 weeks for 13 weeks. At the end of Epoch 1, eligible patients entered Epoch 2 and switched treatment to weekly subcutaneous infusions of Ig20Gly 50–200 mg/kg body weight for 24 weeks (two 12-week treatment periods). Dose adjustments were made during Weeks 1–12 of Epoch 2, if needed, to maintain a target serum total IgG trough level of ≥5 g/l. Infusion training was also performed during Weeks 1–12. Efficacy evaluations were performed during Weeks 13–24 of Epoch 2. At the end of Epoch 2, a subset of up to seven eligible patients then entered Epoch 3 and received Ig20Gly every 2 weeks for 12 weeks at a dose level approximately double that of Epoch 2 (100–400 mg/kg body weight).

### Participants

Patients were eligible for inclusion if they were of Japanese descent and aged 2 years or older at the time of screening, had a documented diagnosis of a form of PID involving antibody formation and requiring immunoglobulin replacement, had received a stable dose of IVIG for at least 3 months prior to screening, had a serum IgG trough level of ≥ 5 g/l at screening, had not had a serious bacterial infection within the 3 months prior to screening, and provided written informed consent. Full details of the inclusion and exclusion criteria for this study are provided in the Supplementary Text 1.

### Endpoints

The primary endpoint of the study was serum total IgG trough levels after weekly administration of Ig20Gly (Epoch 2, Weeks 13–24) and after administration of Ig20Gly every 2 weeks (Epoch 3). For Epoch 2 (Weeks 13–24), PK assessment was intended to be performed in 5–7 patients aged 12 years and older within the last month on weekly Ig20Gly. PK serial sampling started at Epoch 2 Week 21 (± 1 week). The pre-infusion sample (i.e. the trough level of the previous infusion within 1 hour before infusion) was defined as PK Day 0. Serial sampling time points were conducted on Days 1, 3, 5, and 7 (all ± 6 hours, from infusion start time of Day 0). To evaluate if the serum total IgG levels remained stable throughout Epochs 2 and 3 after Ig20Gly stabilization, the geometric mean of the last three serum IgG trough level values at the end of each epoch was chosen for comparison. Only the last three IgG trough values were chosen in Epochs 1 and 3 based on the sampling schedule.

Secondary endpoints included efficacy, safety, and health-related quality of life (HRQoL) measures. Secondary efficacy endpoints included analysis of PK parameters for serum total IgG (Epoch 2), and analysis of serum total IgG trough levels following administration of IVIG every 3 weeks or every 4 weeks (Epoch 1).

Safety endpoints, including occurrence of treatment-emergent adverse events (TEAEs), tolerability events related to infusion (IVIG or Ig20Gly), annual rates of infections (including validated acute serious bacterial infections [VASBIs]), and number of days on antibiotics were also assessed. Infections were defined as any adverse event (AE) with preferred terms from the Medical Dictionary for Regulatory Activities version 24.0 system organ class ‘infections and infestations’.

HRQoL outcomes were assessed using the following questionnaires: Pediatric Quality of Life Inventory (Peds-QL) [18], 36-item Short-Health Survey (SF-36) [19], and 5-Dimension 3-Level EuroQol (EQ-5D-3L) [20]. Treatment satisfaction was measured using the Life Quality Index questionnaire (LQI) [21] and the 9-item Treatment Satisfaction Questionnaire for Medication (TSQM-9). Treatment preference (measured using a treatment preference questionnaire at the last patient visit) and healthcare resource utilization (including hospitalizations, length of stay, acute care visits, and days missed from work/school owing to infection) were also assessed. Full details are provided in Supplementary Text 2.

### Statistical analysis

#### Sample size

A total of 16 patients was planned for inclusion in this study, with 12 patients expected to complete Epoch 2 based on a conservative dropout rate of 25% (based on previous studies). The sample size estimate was based on the small size of the Japanese population with PIDs. The study was not designed for statistical hypothesis testing.

#### Analysis populations

The enrolled set included all screened patients for whom an enrolment number had been assigned and was used for the analysis of patient demographics and baseline clinical characteristics. Baseline was defined as Epoch 1 Visit 1. The all-treated set included all enrolled patients who received at least one dose of study drug (IVIG or Ig20Gly) and was used for all efficacy, safety, tolerability, HRQoL, healthcare resource utilization, and treatment preference outcomes analyses.

The PK analysis set included all enrolled patients who received at least one dose of study drug (IVIG or Ig20Gly), had at least one evaluable serum total IgG concentration value, and had no major protocol deviations or events that would affect the serum IgG concentration analysis results. Analysis of PK data (serum total IgG trough levels and PK profiles) was based on the PK analysis set.

Descriptive statistics were used for all analyses. These included geometric mean and the corresponding two-sided 95% confidence interval (CI) for serum total IgG trough levels; the CIs were for descriptive purposes only. No formal statistical comparison of treatments was performed.

## Results

### Patient disposition and characteristics

**Figure 2.**
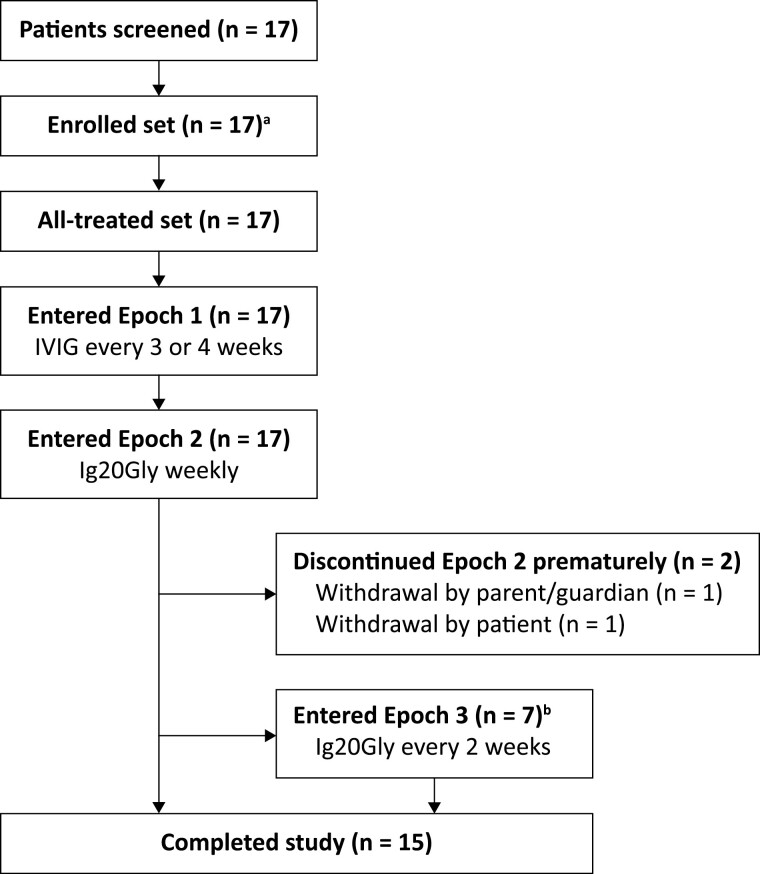
Patient disposition. ^a^Three patients were eliminated in a first screening, but succeeded in re-screening and received the study dose. ^b^Epoch 3 was planned for a subset of up to seven eligible patients. Ig20Gly, immune globulin subcutaneous (human) 20% solution; IVIG, intravenous immunoglobulin.

In total, 17 patients were screened at eight centres in Japan, all of whom were eligible for entry into Epoch 1. All 17 patients progressed to Epoch 2 and a subset of seven patients entered Epoch 3; overall, 15 patients completed the study (i.e. completed Epoch 2 or Epoch 3) (Fig. 2). The two patients who did not complete the study withdrew consent in Epoch 2 (one each by parent/guardian and by the patient); neither withdrawal was due to AEs. Patient demographics and baseline characteristics are presented in Table 1.

**Table 1.** Patient demographics and baseline clinical characteristics

Characteristic	Epochs 1 and 2 (*N* = 17)	Epoch 3 (*N* = 7)
Age, years		
Mean (SD)	31.0 (21.1)	30.3 (20.3)
Median (range)	24.0 (5.0–69.0)	24.0 (5.0–54.0)
Age group, *n* (%)		
<18 years	6 (35.3)	2 (28.6)
≥18 years	11 (64.7)	5 (71.4)
Sex, *n* (%)		
Male	10 (58.8)	4 (57.1)
Female	7 (41.2)	3 (42.9)
Race, *n* (%)		
Asian	17 (100.0)	7 (100.0)
BMI, kg/m^2^		
Mean (SD)	19.9 (3.2)	20.2 (2.8)
Median (range)	20.5 (15.4–26.4)	20.5 (15.5–23.8)
PID diagnosis, *n* (%)		
Common variable immunodeficiency	10 (58.8)	5 (71.4)
X-linked agammaglobulinaemia	7 (41.2)	2 (28.6)
Time since diagnosis, years^a^		
Mean (SD)	5.7 (5.8)	3.8 (4.7)
Median (range)	3.6 (0.7–19.8)	2.0 (0.7–12.1)

All patients eligible for entry into Epoch 1 progressed to Epoch 2, thus the numbers of patients were the same for Epoch 1 and Epoch 2.

^a^ *N* = 14 for Epoch 1 and Epoch 2; precise diagnosis date was unknown for three patients.

BMI, body mass index; PID, primary immunodeficiency disease; SD, standard deviation.

### Serum total IgG trough levels and PK

The geometric mean of serum total IgG trough levels at the end of Epoch 2, after weekly administration of Ig20Gly, was 8.56 g/l (95% CI: 8.03–9.12 g/l, Table 2). At the end of Epoch 3, after administration of Ig20Gly every 2 weeks, the geometric mean of serum total IgG trough levels was similar to that of Epoch 2: 8.39 g/l (95% CI: 7.89–8.91 g/l, Table 2). Similar results were observed using the arithmetic mean and the median of serum total IgG trough levels at the end of each epoch (Table 2). In a secondary endpoint analysis at the end of Epoch 1, the geometric mean for the 3-week dosing interval was slightly lower than that of the 4-week dosing interval: 7.52 g/l (95% CI: 6.42–8.82 g/l) and 8.47 g/l (95%CI: 8.01–8.95), respectively.

**Table 2.** Total serum IgG trough levels at the end of each epoch

Total IgG, g/l	Epoch 1 IVIG 3-week/4-week interval^a^ (*N* = 51)	Epoch 2 Ig20Gly weekly^b^ (*N* = 43)	Epoch 3 Ig20Gly every 2 weeks^c^ (*N* = 18)	Epochs 2 and 3 Ig20Gly^b^^,^^c^ (*N* = 61)
Geometric mean (95% CI)	8.18 (7.71–8.68)	8.56 (8.03–9.12)	8.39 (7.89–8.91)	8.51 (8.11–8.92)
Arithmetic mean (SD)	8.35 (1.68)	8.74 (1.82)	8.45 (1.03)	8.65 (1.62)
Median (range)	7.98 (4.61–10.80)	8.66 (6.06–13.00)	8.31 (6.89–10.00)	8.34 (6.06–13.00)

^a^Data represent measurements of total IgG from Weeks 7, 10, and 13 of the 3-week treatment interval (*n* = 5 patients), and from Weeks 5, 9, and 13 of the 4-week treatment interval (*n* = 12 patients).

^b^Data represent measurements of total IgG from Weeks 17, 21, 25, and EOS/ET within study days 203–261.

^c^Data represent measurements of total IgG from Weeks 5, 9, and 13 (EOS/ET).

CI, confidence interval; EOS, end of study; ET, early termination; Ig20Gly, immune globulin subcutaneous (human) 20% solution; IgG, immunoglobulin G; IVIG, intravenous immunoglobulin; SD, standard deviation.

**Figure 3.**
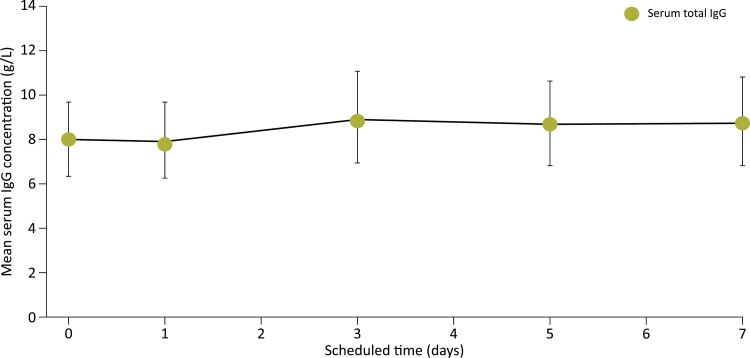
Arithmetic mean serial serum total IgG concentrations Epoch 2 Week 21. IgG, immunoglobulin G.

During Epoch 2, the arithmetic mean PK concentration–time profile for serum total IgG levels remained stable over the entire dosing interval at Week 21 (Figure 3). PK parameters for total IgG in Epoch 2 Week 21 are summarized in Supplementary Table S1. The geometric means (95% CIs) of the area under the concentration–time curve (AUC) for total IgG over a 1-week (168-hour) dosing interval (AUC_0–tau_) and up to last time point (AUC_0–last_), normalized by the Ig20Gly dose/bodyweight, were the same at 58.9 (48.7–71.3) g * day/l. The geometric mean (95% CI) for maximum concentration (*C*_max_) was 9.08 (7.46–11.1) g/l and was 1.93 (1.71–2.19) ml/kg/day for apparent clearance (CL/F). Median (range) time to maximum concentration (*T*_max_) was 71.08 (22.82–168.23) hours.

### Infections

The annual rate of VASBIs during Ig20Gly treatment (Epochs 2 and 3) was 0.11 events per patient per year (99% upper confidence bound: 0.38). One VASBI (bacterial pneumonia) was observed in one patient in Epoch 2 (annual rate in Epoch 2: 0.13). The VASBI resolved with medication, including antibiotics, and without hospitalization. No VASBIs were reported in Epoch 1 or Epoch 3.

The annual rate of all infections during Ig20Gly treatment (Epochs 2 and 3) was 2.04 (95% CI: 1.04–3.56) events per patient. There were 19 infections in 11 patients in Epoch 2 (annual rate in Epoch 2: 2.48 [95% CI: 1.34–4.13]) and no infections were observed in Epoch 3. Of those patients with an infection in Epoch 2, the majority experienced infections that were mild in severity (9 patients); 2 patients experienced moderate infections. The most common infection in Epoch 2 was sinusitis (3 events in 2 patients), followed by conjunctivitis and gastroenteritis (each 2 events in 2 patients). All infections in Epoch 2 were resolved during the study, except one incidence of conjunctivitis (with a duration of ~7.5 months), which was present before the first dose of study drug and thus considered unrelated to Ig20Gly by the investigator. This patient had a history of conjunctivitis prior to study entry.

The annual rate of all infections in Epoch 1 with patients receiving IVIG treatment was 1.65 (95% CI: 0.73–3.15) events per patient. There were seven infections in six patients in Epoch 1, with the most common being nasopharyngitis (two events in two patients).

### Healthcare resource utilization

One hospitalization was recorded during the study of a patient in Epoch 2. This event occurred owing to patient convenience and not because of an illness or an AE. The annualized per-person rate of acute care visits during Ig20Gly treatments (Epochs 2 and 3) was 2.04 (95% CI: 0.78–4.24). There were 18 acute care visits recorded in Epoch 2 (annual rate: 2.35) and 1 visit in Epoch 3 (annual rate: 0.61). There were no emergency room visits recorded at any point during the study. The annualized number of days on antibiotics in Epochs 2 and 3 was 5.38 (95% CI: 2.10–11.02): 5.87 (95% CI: 2.32–11.93) days in Epoch 2 and 3.07 (95% CI: 0.37–10.74) days in Epoch 3.

In Epochs 2 and 3, in total 38 days (22 days in Epoch 2 and 16 in Epoch 3) were recorded in which the patient was not able to attend work/school or to perform normal daily activities because of illness/infection, with a rate of 4.09 (95% CI: 2.02–7.23) days per person-year (Epoch 2 rate: 2.87; Epoch 3 rate: 9.82). Of note, 8 days of hospitalization for one patient after their last visit in Epoch 2 were counted as days not able to attend missed from work/school or to perform normal daily activities in Epoch 3.

In Epoch 1 with patients receiving IVIG, the annualized per-person rate of acute care visits was 1.18 (95% CI: 0.38–2.68; 5 acute care visits recorded). The annualized number of days on antibiotics in Epoch 1 was 4.01 (95% CI: 1.46–8.54) and in total 11 days were recorded in which the patient was not able to attend work/school or to perform normal daily activities, with a rate of 2.60 (95% CI: 1.02–5.30) days per person-year.

### Safety and tolerability

The median dose of Ig20Gly administered was stable in Epoch 2 and Epoch 3: 110.0 mg/kg/week for 168.0 days (range: 85.0–252.0 days) and 108.7 mg/kg/week for 85.0 days (range: 85.0–85.0 days), respectively. Across Epochs 2 and 3 combined, the median number of infusion sites per infusion was 1.2 (range: 1.0–2.0). The median dose of IVIG administered in Epoch 1 was 127.7 mg/kg/week for 91.0 days (range: 90.0–92.0 days). Further details on treatment administration parameters are included in Supplementary Table S2. The median percentage of infusions completed as planned was 100.0% (range: 94.4–100.0%). There was only one infusion interruption in Epoch 2 due to leakage in the infusion system (needle), thus was not related to tolerability. There were no instances of infusion rate reduction or stopped infusions.

Across all epochs the majority of TEAEs, including drug-related TEAEs, were mild and no severe TEAEs were observed during the study (Table 3). Of 108 TEAEs reported across all epochs (excluding infections), 77 events were reported in Epoch 2 and 3 in 15 (88.2%) Ig20Gly-treated patients (Epoch 2: 73 events in 15 patients; Epoch 3: 4 events in 3 patients); of these, 51 events in 12 patients (70.6%) were considered related to the study drug (Table 4). In Epoch 1, 31 events were reported in 9 patients, of which 16 events were considered related or temporally associated to IVIG treatment.

**Table 3.** Summary of TEAEs (including infections) by severity

*n* (%), m	Epoch 1 IVIG 3-week/ 4-week interval (*N* = 17)	Epoch 2 Ig20Gly weekly (*N* = 17)	Epoch 3 Ig20Gly every 2 weeks (*N* = 7)	Epochs 2 and 3 Ig20Gly (*N* = 17)	Overall (*N* = 17)
Any TEAE	11 (64.7), 38	16 (94.1), 92	3 (42.9), 4	16 (94.1), 96	16 (94.1), 134
Related	0 (0.0), 0	12 (70.6), 48	2 (28.6), 3	12 (70.6), 51	12 (70.6), 51
Unrelated	11 (64.7), 38	14 (82.4), 44	1 (14.3), 1	14 (82.4), 45	15 (88.2), 83
Any mild TEAE	11 (64.7), 34	16 (94.1), 88	3 (42.9), 4	16 (94.1), 92	16 (94.1), 126
Related	0 (0.0), 0	12 (70.6), 48	2 (28.6), 3	12 (70.6), 51	12 (70.6), 51
Unrelated	11 (64.7), 34	13 (76.5), 40	1 (14.3), 1	13 (76.5), 41	15 (88.2), 75
Any moderate TEAE	1 (5.9), 4	3 (17.6), 4	0 (0.0), 0	3 (17.6), 4	4 (23.5), 8
Related	0 (0.0), 0	0 (0.0), 0	0 (0.0), 0	0 (0.0), 0	0 (0.0), 0
Unrelated	1 (5.9), 4	3 (17.6), 4	0 (0.0), 0	3 (17.6), 4	4 (23.5), 8

No severe or serious TEAEs were reported. The duration of each study epoch was as follows: Epoch 1 (13 weeks), Epoch 2 (24 weeks), and Epoch 3 (12 weeks).

Ig20Gly, immune globulin subcutaneous (human) 20% solution; IVIG, intravenous immunoglobulin; TEAE, treatment-emergent adverse event.

**Table 4.** Summary of TEAEs (excluding infections)

*n* (%), m	Epoch 1 IVIG 3-week/ 4-week interval (*N* = 17)	Epoch 2 Ig20Gly weekly (*N* = 17)	Epoch 3 Ig20Gly every 2 weeks (*N* = 7)	Epochs 2 and 3 Ig20Gly (*N* = 17)	Overall (*N* = 17)
Any TEAE	9 (52.9), 31	15 (88.2), 73	3 (42.9), 4	15 (88.2), 77	16 (94.1), 108
Related^a^	0 (0.0), 0	12 (70.6), 48	2 (28.6), 3	12 (70.6), 51	12 (70.6), 51
Unrelated	9 (52.9), 31	11 (64.7), 25	1 (14.3), 1	11 (64.7), 26	14 (82.4), 57
Any local TEAE	0 (0.0), 0	10 (58.8), 40	1 (14.3), 2	10 (58.8), 42	10 (58.8), 42
Related^a^	0 (0.0), 0	10 (58.5), 40	1 (14.3), 2	10 (58.8), 42	10 (58.8), 42
Unrelated	0 (0.0), 0	0 (0.0), 0	0 (0.0), 0	0 (0.0), 0	0 (0.0), 0
Any systemic TEAE	9 (52.9), 31	13 (76.5), 33	2 (28.6), 2	13 (76.5), 35	14 (82.4), 66
Related^a^	0 (0.0), 0	4 (23.5), 8	1 (14.3), 1	4 (23.5), 9	4 (23.5), 9
Unrelated	9 (52.9), 31	11 (64.7), 25	1 (14.3), 1	11 (64.7), 26	14 (82.4), 57
Any related or temporally associated TEAE	6 (35.3), 16	14 (82.4), 64	2 (28.6), 3	14 (82.4), 67	15 (88.2), 83
Any TEAE by preferred term, occurring in ≥ 3 patients overall					
Headache Events PPY, *n*	3 (17.6), 7 1.652	3 (17.6), 8 1.043	1 (14.3), 1 0.614	3 (17.6), 9 0.968	4 (23.5), 16 1.182
Injection site swelling Events PPY, *n*	0 (0.0), 0 0.000	3 (17.6), 7 0.913	1 (14.3), 1 0.614	4 (23.5), 8 0.860	4 (23.5), 8 0.591
Injection site erythema Events PPY, *n*	0 (0.0), 0 0.000	3 (17.6), 4 0.522	1 (14.3), 1 0.614	3 (17.6), 5 0.538	3 (17.6), 5 0.369
Stomatitis Events PPY, *n*	3 (17.6), 4 0.944	1 (5.9), 1 0.130	0 (0.0), 0 0.000	1 (5.9), 1 0.108	3 (17.6), 5 0.369

*n* is the number of patients experiencing the event; *m* is the number of events. TEAEs were classified according to MedDRA version 24.0 preferred terms. The duration of each study epoch was as follows: Epoch 1 (13 weeks), Epoch 2 (24 weeks), and Epoch 3 (12 weeks).

^a^All drug-related events were mild in severity.

Ig20Gly, immune globulin subcutaneous (human) 20% solution; IVIG, intravenous immunoglobulin; MedDRA, Medical Dictionary for Regulatory Activities; PPY, per patient-year; TEAE, treatment-emergent adverse event.

Across Epochs 2 and 3, 55% of TEAEs (excluding infection) were local (42 of 77). The most common TEAEs during Ig20Gly treatment were headache (nine events in three patients [17.6%]; 0.968 events per patient-year) and injection site swelling (eight events in four patients [23.5%]; 0.860 events per patient-year) with almost all events occurring during Epoch 2 (Table 4). In Epoch 1 (IVIG treatment), all TEAEs were systemic, the most common of which were headache (seven events in three patients [17.6%]; 1.652 events per patient) and stomatitis (four events in three patients [17.6%]; 0.944 events per-patient). There were no deaths, serious TEAEs, or TEAEs leading to study drug withdrawal or discontinuation that occurred throughout the study, including during treatment with Ig20Gly.

### HRQoL outcomes and treatment satisfaction

Median scores obtained from HRQoL and treatment satisfaction questionnaires were similar when comparing baseline and end of the study, with scores in the upper part of the possible score ranges. Results are summarized in Supplementary Tables 3 and 4.

### Treatment preference

**Figure 4.**
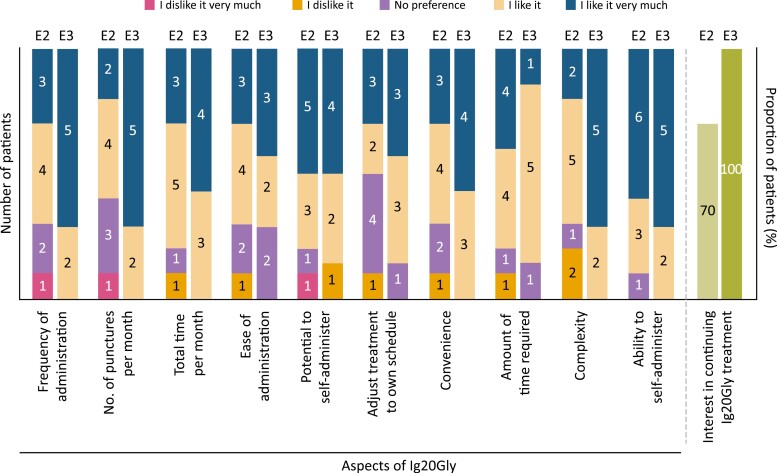
Patient preference and satisfaction with aspects of Ig20Gly administration at the end of Epoch 2 and Epoch 3. E, epoch; Ig20Gly, immune globulin subcutaneous (human) 20% solution.

At the end of the study, most patients (14 of 17) expressed a preference for Ig20Gly over IVIG. Treatment aspects relating to the ability to self-administer and time taken to administer/spent on treatment each month were the highest-rated aspects of Ig20Gly treatment (≥ 80% of respondents rating as ‘I like it’ or ‘I like it very much’) in both Epochs 2 and 3 (Fig. 4). At the end of Epoch 2, seven out of ten respondents expressed an interest in continuing Ig20Gly treatment; at the end of Epoch 3, all respondents (*n* = 7) indicated that they would like to continue.

## Discussion

This study was conducted to evaluate the PK, safety, tolerability, and efficacy of Ig20Gly in Japanese patients with PIDs, the results of which are consistent with the two Ig20Gly pivotal phase 2/3 studies in Europe and in North America [11, 12]. When administered weekly or every 2 weeks, Ig20Gly infusions resulted in serum total IgG trough levels that exceeded a preset, minimum protective trough level (level required for inclusion in this study) of 5 g/l [22], and were similar to those observed following IVIG infusion in Epoch 1. In addition, total IgG concentrations remained stable during the weekly dosing interval in Epoch 2 Week 21.

Consistent with the results demonstrating maintenance of a protective serum IgG trough level, patients in this study had low annual rates of VASBIs and all infections. Only one VASBI was reported during the 24-week Epoch 2 (annual rate: 0.13) and none were reported in the 12-week Epoch 3, resulting in an overall annual rate of VASBIs of 0.11 during Ig20Gly treatment (Epochs 2 and 3 combined). This annualized rate of VASBIs was calculated as higher than those of the European and North American Ig20Gly phase 2/3 studies (0.02 and 0.01, respectively) [11, 12]. However, the sample size was smaller in the current study (17 patients) and the treatment period was shorter (24 weeks) compared with the previous studies (European study: 48 patients for 52 weeks; North American study: 74 patients for 64–68 weeks) [11, 12]. Therefore, it was not feasible to set comparable levels of statistical power or a similar sample size in the current study versus these pivotal trials, limiting the scope for comparison. The annual rate of all infections in Epoch 1 (IVIG treatment) and Epoch 2 were similar, with the Epoch 2 annual rate (2.48 per patient) in line with or lower than those reported in the two pivotal studies [11, 12]. There were no infections observed in Epoch 3 after Ig20Gly administration every 2 weeks. The annual rates of infection were also similar to or slightly lower than reported in a previous clinical study of another 20% SCIG, IgPro20 (L-proline-stabilized 20% human subcutaneous immunoglobulin), in a Japanese population [23]. In the phase 3 study of IgPro20, in which patients received three IVIG infusions at 3–4-week intervals followed by a dose-equivalent switch to weekly SCIG infusions for 12 weeks, the annualized rate of infections was 2.98 per patient (21 patients assessed) [23].

Administration of Ig20Gly both weekly and every 2 weeks was well tolerated and no TEAEs emerged as new safety findings compared with the two pivotal studies [11, 12]. In this study, the median number of infusion sites used per infusion was low: 1.0 for weekly Ig20Gly infusions and 2.0 for bi-weekly infusions. No deaths, serious or severe TEAEs, or TEAEs leading to study drug withdrawal or discontinuation occurred during the study. With respect to tolerability, the median (range) percentage of infusions completed as planned was 100% (94.4–100.0%). Only one infusion interruption occurred during Epoch 2 owing to leakage in the infusion system, and not due to any tolerability findings. Observations that the majority of TEAEs in Epochs 2 and 3 were mild and that headache and infusion site swelling were the most common TEAEs (excluding infections) were consistent with those made in the pivotal studies [11, 12]. The incidence of drug-related local TEAEs was highest in Epoch 2, which is not unexpected following a switch from IVIG to weekly subcutaneous Ig20Gly, and a similar tendency was also seen in the pivotal studies [11, 12]. However, the incidence of local TEAEs decreased over time into Epoch 3 (Ig20Gly administered every 2 weeks), consistent with other observations [24].

With respect to HRQoL measures and treatment satisfaction, scores recorded at the end of the study (Epochs 2 and 3) were generally similar to those observed at baseline and were in the upper part of the possible score ranges. These results suggest that HRQoL and treatment satisfaction of patients were maintained after switching from IVIG to Ig20Gly, although the short treatment period would make it difficult to determine changes over time. Overall, treatment preference favoured Ig20Gly over IVIG administration. These results were consistent with the combined data from the two pivotal studies, with respect to the fact that the median scores from HRQoL and treatment satisfaction measures remained in the upper part of the possible score range, and patients preferred Ig20Gly to IVIG [25]. These results also complement real-world data in which patients expressed overall satisfaction with Ig20Gly at 12 months post-initiation, with all respondents indicating they would like to continue Ig20Gly [15].

There were some limitations of this study that should be noted. First, the sample size for the study was small because of the small numbers of eligible patients in the Japanese population. As a result, statistical analyses were descriptive rather than inferential and should be interpreted with caution owing to statistical significance not being confirmed. Nonetheless, for the objectives of this study, and based on feasibility and the sponsor’s experience with IgG products, the sample size was considered adequate for providing reliable estimates of trough levels and other endpoints. Second, the long-term safety and efficacy of Ig20Gly in Japanese patients with PIDs is yet to be confirmed. This will be addressed in an ongoing extension study (NCT04842643).

SCIG is commonly dosed weekly. Based on PK studies, it is feasible to dose SCIG every 2 weeks to maintain a protective serum IgG trough level [26]; however, there has been a limited amount of data on two-weekly dosing. In this phase 3, open-label study, the primary endpoint was met: Ig20Gly infusions administered weekly or every 2 weeks maintained protective serum total IgG trough levels in adult and paediatric Japanese patients with PIDs. The low annual rates of infections were consistent with serum total IgG trough levels being maintained above a protective threshold and both Ig20Gly dosing regimens were well tolerated, with a low number of infusion sites, and no new or unexpected safety findings identified. HRQoL assessments and patient-reported outcomes of treatment satisfaction and preference showed that patients appreciated the treatment flexibility and convenience offered by weekly and bi-weekly Ig20Gly preparations, with overall treatment preference favouring Ig20Gly over IVIG.

## Supplementary Material

ltae001_suppl_Supplementary_Material

## Data Availability

Data pertaining to this study and the associated study protocol may be found at ClinicalTrials.gov (identifier: NCT04346108) and jRCT.niph.go.jp (identifier: jRCT2080225067). The data sets, including the redacted study protocol, redacted statistical analysis plan, and individual participant data supporting the results reported in this article, will be made available within 3 months from the initial request to researchers who provide a methodologically sound proposal. The data will be provided after their de-identification, in compliance with applicable privacy laws, data protection, and requirements for consent and anonymization.

## Abbreviations

AE: Adverse event
AUC: Area under curve
BMI: Body mass index
CI: Confidence interval
CL/F: Apparent clearance
E: Epoch
EOS: End of study
ET: Early termination
EQ-5D-3L: 5-Dimension 3-Level EuroQol
GM: Geometric mean
HRQoL: Health-related quality of life
Ig20Gly: Immune globulin subcutaneous (human) 20% solution
IgG: Immunoglobulin G
IgPro20: L-proline-stabilized 20% human subcutaneous immunoglobulin
IgRT: Immunoglobulin replacement therapy
IVIG: Intravenous immunoglobulin
LQI: Life Quality Index
MedDRA: Medical Dictionary for Regulatory Activities
Peds-QL: Paediatric Quality of Life Inventory
PID: Primary immunodeficiency disease
PK: Pharmacokinetics
PPY: Per person-year
SCIG: Subcutaneous immunoglobulin
SD: Standard deviation
SF-36: 36-Item Short-Form Health Survey
TEAE: Treatment-emergent adverse event
TSQM-9: 9-item Treatment Satisfaction Questionnaire for Medication
VASBI: Validated acute serious bacterial infections.
