# Supplementary material for: Pharmacokinetics, safety, and efficacy of 20% subcutaneous immunoglobulin (Ig20Gly) administered weekly or every 2 weeks in Japanese patients with primary immunodeficiency diseases: a phase 3, open-label study
Source: Immunother Adv. 2024 Mar 1;4(1):ltae001. doi: 10.1093/immadv/ltae001 (PMC10953624; doi:10.1093/immadv/ltae001)
Supplement: ltae001_suppl_Supplementary_Material [file ltae001_suppl_supplementary_material.pdf]

Supplementary material

Pharmacokinetics, safety, and efficacy of 20% subcutaneous immunoglobulin (Ig20Gly) administered weekly or every 2 weeks in Japanese patients with primary immunodeficiency diseases: a phase 3, open-label study

Hirokazu Kanegane,[1] Akifumi Endo,[1] Satoshi Okada,[2] Hidenori Ohnishi,[3] Masataka Ishimura,[4] Ryuta Nishikomori,[5] Kohsuke Imai,[6] Shigeaki Nonoyama,[6] Hideki Muramatsu,[7] Taizo Wada,[8] Atsushi Kuga,[9] Ko Sakamoto,[9] Sharon Russo-Schwarzbaum,[10] Liang-Hui Chu,[11] Barbara McCoy,[10] Zhaoyang Li,[11] Leman Yel[11,12,*]

[1]Department of Child Health and Development, Tokyo Medical and Dental University, Tokyo, Japan

[2]Department of Pediatrics, Hiroshima University Graduate School of Biomedical and Health Sciences, Hiroshima, Japan

[3]Graduate School of Medicine, Gifu University, Gifu, Japan

[4]Department of Pediatrics, Kyushu University Hospital, Fukuoka, Japan

[5]Department of Pediatrics and Child Health, Kurume University Hospital, Fukuoka, Japan

[6]Department of Pediatrics, National Defense Medical College, Saitama, Japan

[7]Department of Pediatrics, Nagoya University Hospital, Aichi, Japan

[8]Institute of Medical, Pharmaceutical and Health Sciences, Kanazawa University Hospital, Ishikawa, Japan

[9]Takeda Pharmaceutical Company Limited, Osaka, Japan

[10]Baxalta Innovations GmbH, a Takeda company, Vienna, Austria

[11]Takeda Development Center Americas, Inc., Cambridge, MA, USA

[12]Present address: Department of Medicine, University of California Irvine, Irvine, CA, USA

*Correspondence: Department of Medicine, University of California Irvine, C240 Medical Sciences 1, Irvine, CA 92697, USA. Email: lyel@uci.edu

Supplementary Text 1

Patients who met any of the following criteria were included in the study.

- Were of Japanese descent (defined as born in Japan and having Japanese parents and Japanese maternal and paternal grandparents).
- Had a documented diagnosis of a form of primary immunodeficiency disease involving antibody formation and requiring gammaglobulin replacement, as defined according to the International Union of Immunological Societies Committee 2017 [1]. The diagnosis had to have been confirmed by the Medical Director (sponsor's study physician) prior to treatment with intravenous immunoglobulin (IVIG).
- Were aged 2 years or older at the time of screening.
- Written informed consent was provided by either the patient or the patient's legally authorized representative prior to any study-related procedures and study product administration.
- Had been receiving a consistent dose of IVIG over a period of ≥ 3 months prior to screening equivalent to ~200–600 mg/kg body weight per 3–4-week period, according to the product package insert.
- Had a serum IgG trough level of ≥ 5 g/l at screening.
- Had not had a serious bacterial infection within the 3 months prior to screening.
- Were willing and able to comply with the requirements of the protocol.

Patients who met any of the following criteria were excluded from the study.

- Had a known history of, or was positive at screening for, one or more of the following: hepatitis B surface antigen, polymerase chain reaction for hepatitis C virus, polymerase chain reaction for human immunodeficiency virus type 1 or 2.
- Abnormal laboratory values at screening meeting any one of the following criteria (abnormal tests may have been repeated once to determine if they were persistent):
    - Persistent alanine aminotransferase and aspartate aminotransferase > 2.5 times the upper limit of normal for the testing laboratory.
    - Persistent severe neutropenia (defined as an absolute neutrophil count ≤ 500/mm^3).
- Had presence of renal function impairment defined by an estimated glomerular filtration rate < 60 ml/min/1.73 m^2.
- Had been diagnosed with or had a malignancy (other than adequately treated basal cell or squamous cell carcinoma of the skin or carcinoma *in situ* of the cervix), unless the disease-free period prior to screening exceeded 5 years.

- Was receiving anti-coagulation therapy or had a history of thrombotic episodes (including deep vein thrombosis, myocardial infarction, cerebrovascular accident, pulmonary embolism) within 12 months prior to screening, or a history of thrombophilia.
- Had abnormal protein loss (protein-losing enteropathy, nephrotic syndrome).
- Had anaemia that would preclude phlebotomy for laboratory studies according to standard practice at the study site.
- Had an ongoing history of hypersensitivity or persistent reactions (urticaria, breathing difficulty, severe hypotension, or anaphylaxis) following IVIG, subcutaneous immunoglobulin, and/or immune serum globulin infusions.
- Had immunoglobulin A (IgA) deficiency (IgA levels < 0.07 g/l), known anti-IgA antibodies, and a history of hypersensitivity.
- Was on preventative (prophylactic) systemic antibacterial antibiotics at doses sufficient to treat or prevent bacterial infections and could not stop these antibiotics at the time of screening.
- Had an active infection and was receiving antibiotic therapy for the treatment of infection at the time of screening.
- Had a bleeding disorder, or a platelet count < 20 000/µl, or, in the opinion of the investigator, would have been at significant risk of increased bleeding or bruising as a result of subcutaneous therapy.
- Had total protein levels > 9 g/dl, or myeloma, or macroglobulinaemia, or paraproteinaemia.
- Women of childbearing potential who met any one of the following criteria:
    - Presented with a positive pregnancy test
    - Was breastfeeding
    - Intended to begin nursing during the course of the study
    - Did not agree to employ adequate birth control measures (e.g. intrauterine device, diaphragm or condom (for male partner) with spermicidal jelly or foam, or birth control pills/patches) throughout the course of the study.
- Had participated in another clinical study and had been exposed to an investigational product or device within 30 days prior to study enrolment.
- Was scheduled to participate in another non-observational (interventional) clinical study involving an investigational product or device during the course of the study (exception: entering the extension [ClinicalTrials.gov identifier: NCT04842643] of the current study).
- Had severe dermatitis that would preclude adequate sites for safe product administration.

Supplementary Text 2

- The Pediatric Quality of Life Inventory (Peds-QL) was completed only in patients aged 2–13 years (the observers of patients aged 2–7 years and 8–13 years were the parent/guardian and patient themself, respectively) and has a Total Scale Score of 0–100, in which higher scores indicate better health-related quality of life (HRQoL).
- The 36-item Short-Health Survey (SF-36v2) was only completed in patients aged 14 years and older and is reported as summary scores ranging from 0 to 100, in which higher scores indicate better health status.
- For the 5-Dimension 3-Level EuroQol (EQ-5D-3L) questionnaire, the observer of patients aged 2–11 years was the parent/guardian and the observer of patients aged 12 years and older was the patient themself. The EQ-5D-3L health state index score ranges from 0 to 1, with higher scores indicative of better health status (0 is a health state equivalent to death and 1 is equivalent to perfect health).
- Life Quality Index questionnaire (LQI) factor scores range from 0 to 100, in which higher scores indicate better health status.
- The 9-item Treatment Satisfaction Questionnaire for Medication (TSQM-9) domain scores (Effectiveness, Convenience, Global Satisfaction) range from 0 to 100, in which higher scores indicate better satisfaction for that domain.
  For the treatment preference questionnaire, the observer of patients aged 2–13 years was the parent/guardian and the observer of patients aged 14 years or older was the patient themself.

Supplementary Table 1. Pharmacokinetic parameters for total IgG in Epoch 2 Week 21 (*n* = 7)

	**Geometric mean (95% CI)**	**Median (range)**
**$AUC_{0–tau}$, g*day/l**	58.9 (48.7–71.3)	55.1 (46.8–79.6)
**$AUC_{0–tau}$/(dose/weight), (g*day/l)/(g/kg)**	517 (457–586)	514 (408–611)
**$AUC_{0–last}$, g*day/l**	58.9 (48.7–71.3)	55.1 (46.8–79.6)
**$AUC_{0–last}$/(dose/weight), (g*day/l)/(g/kg)**	517 (457–586)	514 (408–611)
**C_{max}, g/l**	9.08 (7.46–11.1)	8.08 (7.08–11.9)
**C_{min}, g/l**	7.45 (6.17–8.99)	6.97 (6.02–11.0)
**T_{max}, h**	NA (NA–NA)[a]	71.08 (22.82–168.23)
**CL/F, ml/kg/day**	1.93 (1.71–2.19)	1.94 (1.64–2.45)

[a]Geometric mean and 95% CI of geometric mean are not estimated for T_{max}.

AUC, area under the curve; CI, confidence interval; CL/F, apparent clearance; C_{max}, maximum concentration; C_{min}, minimum concentration; IgG, immunoglobulin G; NA, not applicable; tau, 24 hours; T_{max}, time to maximum concentration.

Supplementary Table 2. Treatment administration parameters

	**Epoch 1 IVIG 3-week/ 4-week interval (*N* = 17)**	**Epoch 2 Ig20Gly weekly (*N* = 17)**	**Epoch 3 Ig20Gly every 2 weeks (*N* = 7)**	**Epochs 2 and 3 Ig20Gly (*N* = 17)**	**Overall (*N* = 17)**
Number of infusions, median (range)					
Per patient	4.0 (4.0–5.0)	24.0 (12.0–36.0)	6.0 (6.0–6.0)	24.0 (12.0–36.0)	29.0 (16.0–41.0)
Per patient-year	16.1 (15.9–20.1)	51.9 (48.2–52.5)	25.8 (25.8–25.8)	51.6 (43.1–52.2)	37.1 (33.2–43.7)
Per month[a]	1.3 (1.3–1.7)	4.3 (4.0–4.4)	2.2 (2.1–2.1)	4.3 (3.6–4.3)	3.1 (2.8–3.6)
Number of infusion sites, median (range)					
Per infusion[b]	1.0 (1.0–1.0)	1.0 (1.0–2.0)	2.0 (2.0–2.0)	1.2 (1.0–2.0)	1.2 (1.0–1.9)
Per month[c]	1.3 (1.3–1.7)	4.3 (4.0–8.7)	4.3 (4.3–4.3)	4.3 (4.0–8.6)	3.6 (2.8–6.2)
Duration of infusion, minutes[d]					
n infusions	73	398	42	440	513
Median (range)	150.0 (87.0–232.0)	35.0 (13.0–116.0)	64.5 (30.0–205.0)	36.0 (13.0–205.0)	39.0 (13.0–232.0)
Maximum infusion rate per site, ml/h					
n infusions	73	533	84	617	690
Median (range)	100.0 (45.0–200.0)	34.0 (13.0–76.0)	38.0 (23.0–38.0)	35.0 (13.0–76.0)	38.0 (13.0–200.0)
Infusion volume per site, ml					
n infusions	73	533	84	617	690
Median (range)	200.0 (40.0–300.0)	17.0 (8.5–32.0)	25.5 (16.5–52.5)	19.0 (8.5–52.5)	19.0 (8.5–300.0)

[a]Number of infusions per month is calculated as (total number of infusions during the corresponding period / days of exposure) × 30.4 days per month.

[b]Number of infusion sites per infusion is calculated as (total number of infusion sites during the corresponding period) / (total number of infusions).

[c]Number of infusion sites per month is calculated as (total number of infusion sites) / (duration in treatment [days] / 30.4 days per month).

[d]Duration of infusion is calculated as stop time of infusion – start time of infusion.

Ig20Gly, immune globulin subcutaneous (human) 20% solution; IVIG, intravenous immunoglobulin.

Supplementary Table 3. HRQoL outcomes

**Assessment**	**Outcome observer**	
**Peds-QL score, median (range)**	**Parent/guardian[a] (*n* = 2)**	**Patient[b] (*n* = 4)**
Epoch 1 Visit 1 (baseline)	88.0 (78.3 –97.8)	88.6 (72.8–98.9)
Epoch 2 Visit 1	97.8 (96.7–98.9)	81.5 (59.8–98.9)
Epochs 2 and 3 EOS/ET	91.3 (87.0–95.7)	98.4 (69.6–98.9)
**SF-36v2 score, median (range)**	**–**	**Patient[c] (*n* = 11)**
**Physical component**		
Epoch 1 Visit 1 (baseline)	–	54.1 (38.1–57.6)
Epoch 2 Visit 1	–	53.6 (37.0–60.4)
Epochs 2 and 3 EOS/ET	–	48.7 (38.7–60.0)
**Mental component**		
Epoch 1 Visit 1 (baseline)	–	52.0 (34.1–61.1)
Epoch 2 Visit 1	–	52.9 (33.4–59.5)
Epochs 2 and 3 EOS/ET	–	51.6 (43.9–72.0)
**EQ-5D-3L health state index score, median (range)**	**Parent/guardian[d] (*n* = 4)**	**Patient[e] (*n* = 13)**
Epoch 1 Visit 1 (baseline)	0.808 (0.768–0.848)	0.848 (0.710–0.848)
Epoch 2 Visit 1	0.848 (0.607–0.848)	0.848 (0.724–0.848)
Epochs 2 and 3 EOS/ET	0.848 (0.848–0.848)	0.848 (0.804–0.848)

[a]Observer of patients aged 2–7 years.

[b]Observer of patients aged 8–13 years.

[c]The SF-36v2 was only completed in patients aged 14 years and older and the observer was the patients themselves.

[d]Observer of patients aged 2–11 years.

[e]Observer of patients aged 12 years and older.

EOS/ET, end of study/early termination; HRQoL, health-related quality of life; Peds-QL, Pediatric Quality of Life Inventory; SF-36v2, 36-item Short-Form Health Survey – version 2.

Supplementary Table 4. Treatment satisfaction

Assessment	Outcome observer	
**LQI score, median (range)**	**Parent/guardian[a] (*n* = 6)**	**Patient[b] (*n* = 11)**
**Treatment interference**		
Epoch 1 Visit 1 (baseline)	76.4 (66.7–100.0)	83.3 (55.6–100.0)
Epoch 2 Visit 1	70.8 (58.3–97.2)	86.1 (41.7–97.2)
Epochs 2 and 3 EOS/ET	84.7 (44.4–100.0)	83.3 (33.3–100.0)
**Therapy-related problems**		
Epoch 1 Visit 1 (baseline)	83.3 (54.2–100.0)	79.2 (58.3–100.0)
Epoch 2 Visit 1	87.5 (62.5–100.0)	75.0 (37.5–100.0)
Epochs 2 and 3 EOS/ET	77.1 (66.7–100.0)	83.3 (54.2–100.0)
**Therapy setting**		
Epoch 1 Visit 1 (baseline)	77.9 (50.0–100.0)	77.8 (38.9–100.0)
Epoch 2 Visit 1	66.7 (55.6–100.0)	88.9 (33.3–100.0)
Epochs 2 and 3 EOS/ET	83.3 (50.0–100.0)	77.8 (50.0–100.0)
**Treatment costs**		
Epoch 1 Visit 1 (baseline)	70.8 (33.3–100.0)	58.3 (25.0–83.3)
Epoch 2 Visit 1	45.8 (33.3–91.7)	50.0 (16.7–100.0)
Epochs 2 and 3 EOS/ET	62.5 (50.0–100.0)	66.7 (33.3–100.0)
**TSQM-9 score, median (range)**	**Parent/guardian[c] (*n* = 6)**	**Patient[d] (*n* = 11)**
**Global satisfaction**		
Epoch 1 Visit 1 (baseline)	78.6 (50.0–92.9)	71.4 (42.9–100.0)
Epoch 2 Visit 1	78.6 (42.9–85.7)	78.6 (50.0–100.0)
Epochs 2 and 3 EOS/ET	78.6 (64.3–100.0)	85.7 (0.0–100.0)
**Effectiveness**		
Epoch 1 Visit 1 (baseline)	69.4 (44.4–83.3)	66.7 (44.4–100.0)
Epoch 2 Visit 1	66.7 (61.1–77.8)	77.8 (44.4–100.0)

Epochs 2 and 3 EOS/ET	75.0 (66.7–100.0)	77.8 (50.0–100.0)
**Convenience**		
Epoch 1 Visit 1 (baseline)	66.7 (44.4–83.3)	61.1 (27.8–100.0)
Epoch 2 Visit 1	66.7 (55.6–94.4)	61.1 (44.4–100.0)
Epochs 2 and 3 EOS/ET	66.7 (44.4–83.3)	77.8 (44.4–100.0)

[a]Observer of patients aged 2–13 years.

[b]Observer of patients aged 14 years and older.

[c]Observer of patients aged 2–12 years.

[d]Observer of patients aged 13 years and older.

EOS/ET, end of study/early termination; LQI, Life Quality Index; TSQM-9, 9-item Treatment Satisfaction Questionnaire for Medication.

Reference

1. Picard C, Bobby Gaspar H, Al-Herz W et al. International Union of Immunological Societies: 2017 primary immunodeficiency diseases committee report on inborn errors of immunity. *J Clin Immunol* 2018; 38(1):96–128. https://doi.org/10.1007/s10875-017-0464-9
